# Results of the national organised colorectal cancer screening program with FIT in Paris

**DOI:** 10.1038/s41598-018-22481-9

**Published:** 2018-03-07

**Authors:** Anna Pellat, Jacques Deyra, Romain Coriat, Stanislas Chaussade

**Affiliations:** 1 Gastroenterology and digestive oncology unit, Cochin teaching Hospital, Paris Descartes University, AP-HP, Paris, France; 2 ADECA 75, Paris, France

## Abstract

In France, colorectal cancer (CRC) benefits from a nationwide screening program. The faecal immunochemical test (FIT) is being used since April 2015. The test is recommended in asymptomatic patients followed by a colonoscopy if positive for identification and treatment of colorectal lesions. We investigate the CRC national organised screening program using FIT in Paris. We performed a retrospective observational study, collecting data from the screening program in Paris using the ADECA75 database. Rates of participation, numbers of positive FIT, detection rates and positive predictive values (PPV) for advanced adenomas (AA) and/or CRC were determined. Between 01/01/2016 and 30/06/2017, 620.227 Parisians were eligible and 409.340 were invited to participate to the program. A total of 88.796 participants (23%) performed the test with 3.839 positive tests (4.3%). In the positive test population, 2.706 out of 3.839 individuals (70.5%) performed the required colonoscopy with available reports. Histology reports were only available for 2.401 participants (88,7%). Regarding lesions, 733 (30,5%) and 205 patients (8.5%) had AA and CRC, respectively. Over 18 months of screening with FIT in Paris, the PPV is in line with expected results while the participation rate is below European recommendations.

## Introduction

Colorectal cancer (CRC) is the 3rd most common cancer in France with an incidence of more than 40.000 in 2012 (www.e-cancer.fr). It is the second cause of death by cancer in France and benefits from an organised nationwide screening program in order to identify asymptomatic individuals with advanced adenomas (AA) and/or (early) cancer. It aims to reduce morbidity and CRC-related mortality. The screening program developed in France was based on a two steps program: first the use of a faecal occult blood test (FOBT) and then, if positive, the performance of a colonoscopy. This program was started in 2007 using the guaiac-based FOBT Hemoccult®: 269.291 of these tests were performed in Paris between 2007 and April 2015. This screening method is based on the oxidation of guaiac by a hydrogen peroxide reaction with haemoglobin (Hb); it requires three stool samples for analysis. It was recommended by guidelines and supported by strong data^1^.

Various factors are determinant of the effectiveness of a screening program such as the cost, the compliance to the test and the sensitivity and specificity of diagnosis. Guaiac-based FOBT have been criticised for their fairly low sensitivity and their lack of specificity for human Hb. Regarding Hemoccult®, positivity rate and sensitivity for detection of CRC are about 2%, and 40% respectively^2,3^.

Therefore, based on its lack of specificity, Hemoccult® was replaced by the faecal immunological test for haemoglobin (FIT; OC sensor®) in April 2015. It detects small amounts of blood in stool samples using antibodies targeting Hb. The FIT test, performed on one sample of stool, showed better participation rates^3,4^. Meantime, the sensitivity of the detection of colorectal neoplasia was estimated to 61.3%^2,5,6^. In countries of northern of Europe, such as the Netherlands, a colonoscopy is performed in 83% of patients with a positive FIT^7^. It has been showed recently in the East region of France that 30% of the population participated to the CRC screening program with FIT^8,9^. In 2014, the Parisian population regrouped 2.220.445 individuals according to the “*Institut National de la Statistique et des Etudes Economiques”* (INSEE) (www.insee.fr). A total of 707.518 individuals were aged between 45 and 74 years and 53% were female. Therefore, about 40% of Parisians were eligible for participation to the CRC screening program in 2014. European guidelines recommend 45% of participation to the test in order to show its benefit^10^. Our work describes the results of the first 18 months of the Parisian organised CRC screening program with FIT.

## Methodology

We performed a retrospective observational study in the Paris area. Data was extracted from the centralised ADECA75 registry. We screened the population of patients in Paris who performed a FIT between the 01/01/2016 and 30/06/2017. We did not include results from 2015 in our analysis because of the difficulties met when launching the new program, (delay in sending new invitations, in distribution of new tests, and in information to physicians) resulting in incorrect data between April and December 2015. To compensate for this delay, many invitations were sent only in 2016, resulting in an important number of reminders.

### Paris CRC screening program

The CRC screening program in Paris was centrally managed by the ADECA75 office which invited the eligible population. The target population regrouped asymptomatic adults aged 50 to 74 years’ old, attached to the Parisian health insurance (department 75) and not following an individual CRC screening program. The target population in Paris during this period of time regrouped 620.227 individuals aged between 50 and 74 years’ old (about 28% compared to the Parisian population in 2014).

Invitations for participation to the CRC screening program were sent by mail from the ADECA75 office to the target population. Individuals were invited to consult their general practitioner (GP) to retrieve the OC Sensor®. The invitation was sent every 2 years. If invitees did not receive the invitation they could ask for it online or call an attributed number. If patients did not respond after a first mail, a reminder was sent after 90 days. A second reminder could be sent after 120 days if there was still a lack of answer. After invitation, patients could be excluded for medical reasons (death, individual screening program…), change in address of residence or performance of a colonoscopy in the past 5 years. Tests were then distributed freely to patients by their GP or specialists. Analysis was performed on one sample of stool returned by mail in a prepaid envelope by participants. When the test was negative, patients stayed in the screening program and received a new invitation 2 years later. If the test was positive, meaning over 30 µgHb/g of stool, they were advised to perform a colonoscopy for detection and/or treatment of potential colorectal lesions and addressed to a gastroenterologist (GI). Participants whose sample was not assessable (outdated test, error in filling out the form…) were sent a new test. Individuals who did not respond to the invitation were labelled as non-responders. In Paris during this time, 2.558 physicians prescribed the OC Sensor®, including 2388 GP (93,3%). In total, 4.325 GP were registered in the area, 30% being practice-oriented GP: about 55% of GP prescribed the OC Sensor®.

Colonoscopy was the standard diagnostic and/or therapeutic exam after a positive test. When the colonoscopy was performed, the ADECA75 office recovered colonoscopy and pathology reports when available. If colonoscopy reports could not be recovered, patients’ results could not be registered in the database. All colonoscopies were performed by 307 GI with a majority working in private practice (71,8%). A few of these physicians worked both in the hospital and in private practice (3,4%).

### Outcomes and analyses

Data was collected to assess participation rates to FIT and positivity rates in the target population. We also evaluated the colonoscopy participation rate, detection rates and positive predictive values (PPV) for colorectal lesions.

The FIT participation rate was defined as the number of persons performing the test (1 stool sample) divided by the number of persons invited by ADECA75 minus the excluded population. The positivity rate was defined by the number of patients with a result at or above 30 µgHb/g of stool (recommended threshold) divided by the number of participants with an assessable test. The participation rate for colonoscopy was defined by the number of patients performing a colonoscopy exam with available reports divided by the number of patients with a positive FIT. Main colorectal lesions described in our work were polyps (hyperplasic and adenomas) and CRC. Polyps with a risk of transformation, or advanced adenomas, were defined as adenomas with size of 10 mm and/or larger, and/or with histology showing villous component or high-grade dysplasia. Festoon polyps were not described in our work. The detection rate was defined as the proportion of persons with colorectal lesions detected during colonoscopy per 1.000 screened persons with an assessable FIT. PPV were calculated as the number of patients with colorectal lesions divided by the number of patients who underwent a colonoscopy with available pathology reports. The false-positive rate was defined as the number of patients with a normal colonoscopy divided by the number of patients with a positive test.

### Data availability

The datasets generated during and/or analysed during the current study are available from the corresponding author on reasonable request.

### Ethics

The Ethical Review Committee for publications of the Cochin University Hospital (CLEP) has examined the research and found it conformed to generally accepted scientific principles and research ethical standards and in conformity with the laws and regulations of the country in which the research experiment was performed. CLEP Decision N°: AAA-2017-06008.

## Results

### Participation and positivity rates for FIT

In Paris between 01/01/2016 and 30/06/2017, 620.227 Parisians were eligible for the CRC screening program (28%) and 409.340 received an invitation by mail for FIT. Then, 468.103 individuals received a second invitation. There were 23.484 patients excluded. The main cause for exclusion was the performance of a colonoscopy in the past 5 years (70,5%). A total of 88.796 tests were performed resulting in a participation rate to FIT of 23%. The majority of tests were delivered by GP: 2.388 out of the 2.588 physicians who prescribed the test during this time (93,3%).

**Figure 1:**
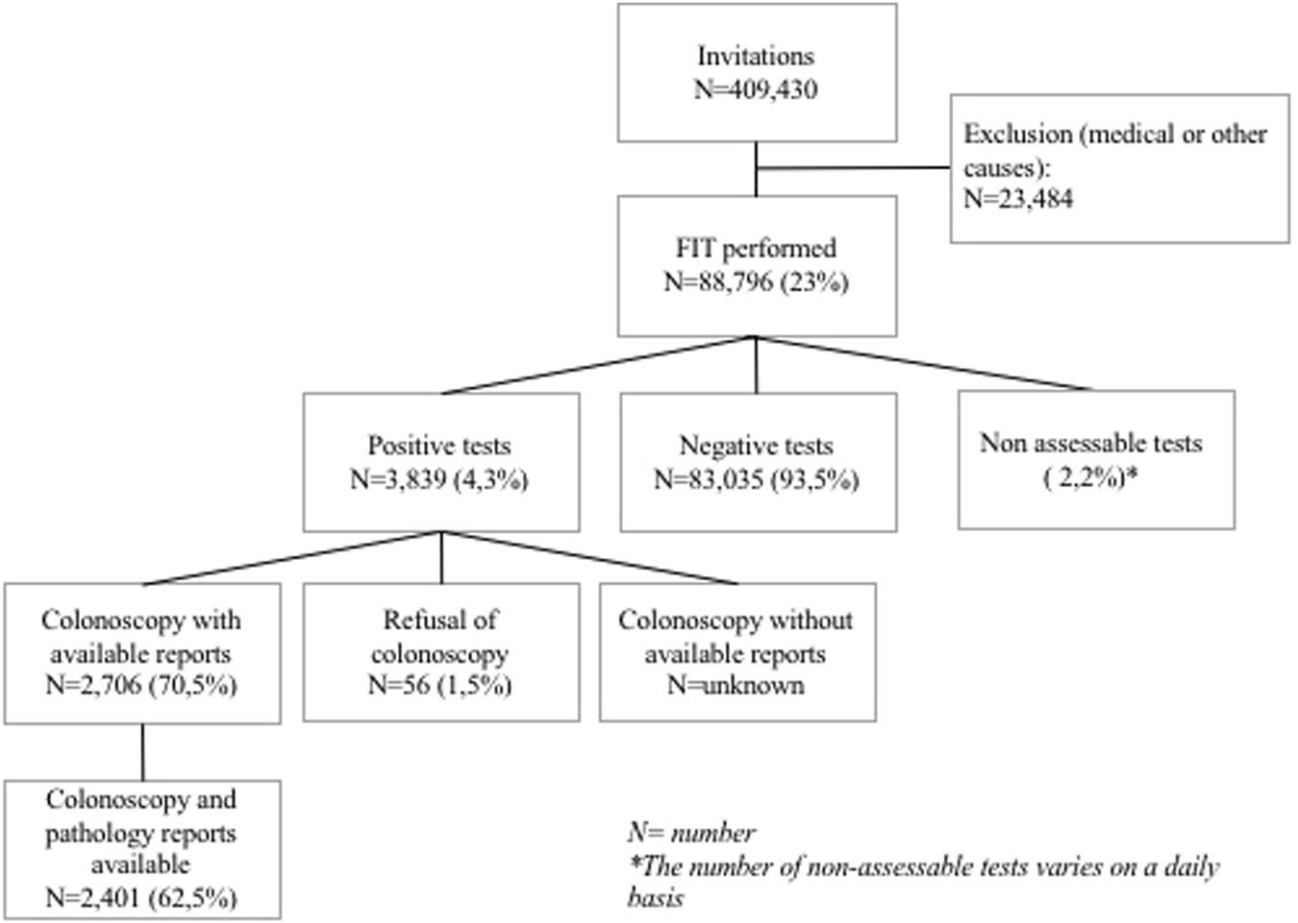
Flow chart of the Parisian population participating in the organised CRC screening program between 01/01/2016 and 30/06/2017.

There were 3.839 positive and 83.035 negative tests, so a positivity rate of 4,3%. The rest of the tests were non-assessable. In our population, the rate of non-assessable tests was at least 2,2% (Fig. 1). Median and mean values of positivity for all positive tests in our population were 69,6 and 98,2 μgHb/g of stool respectively.

### Participation rate for colonoscopy

After a positive test, the rate of participation for a colonoscopy with available reports was 70,5% (2706 out of 3839 participants). Finally, only 2.401 out of 3.839 patients (62,5%) participants had both colonoscopy and pathology reports available. The number of patients who performed a colonoscopy without available reports in the ADECA75 database is unknown. Fifty-six patients refused to perform a colonoscopy after a positive test (1,5%).

In our population, mean time to colonoscopy after a positive test was 74,5 days. Three hundred and seven GI performed these exams with a mean of 7,1 colonoscopies by physician. The majority of colonoscopies were performed in private practice (71,8%).

### Detection rates and PPV values

Despite of a positive test, 551 out of 2.401 participants had a normal colonoscopy (PPV = 22,9%). The false positive rate was 14,4%. A total of 2.401 colorectal lesions were encountered including 386 adenomas (PPV = 16,1%), 733 AA (PPV = 30,5%) and 205 CRC (PPV = 8,5%) (Table 1**)**.

**Table 1:** Predictive positive values (PPV) and detection rates for encountered colorectal lesions after a positive FIT for patients with available colonoscopy and pathology reports (2.401 participants).

**Type of encountered lesions**	**N**	**PPV (%)**	**Detection rates**
Normal colonoscopy	551	22,9	—
IBD	7	0,3	—
Other benign colorectal lesions	353	14,7	—
Hyperplasic polyps	166	6,9	—
Adenomas	386	16,1	4,3
Advanced adenomas	733	30,5	8,3
CRC	205	8,5	2,3
Total	2401	100	—

^*^N: number, PPV: positive predictive value, IBD: inflammatory bowel disease, CRC: colorectal cancer.

Other lesions regroup: haemorrhoids, and diverticula.

Detection rates were defined as the proportion of persons with colorectal lesions detected during colonoscopy per 1.000 screened persons with an assessable FIT.

PPV were calculated as the number of patients with colorectal lesions divided by the number of patients who underwent a colonoscopy with available pathology reports.

Detection rates in our population for adenomas, AA and CRC were 4,3, 8,3 and 2,3 for 1.000 persons, respectively.

## Discussion

In the Paris department over the past 18 months, 23% of invitees participated to the CRC screening program with FIT. PPV for detection of polyps at risk of transformation and CRC were 30,5% and 8,5% respectively.

This work collected data from a large population and is the first to describe the outcomes of the CRC screening program with FIT in the Parisian area. Also, it was managed by a centralised computer database from the ADECA 75 office.

The participation rate to FIT was 23% which is lower than the expected 45% defined by European guidelines. Nevertheless, our results do not reflect the behaviour of the global French population. Indeed, the CRC screening program in France is managed by each department with some variabilities. Parisians are known for their lower rates of participation because of a higher number of colonoscopies performed as an individual screening program. For our population, the major cause of exclusion was the recent performance of a colonoscopy exam (70,5%). This low rate of participation rate could also be related to the decentralised management of the French CRC screening program, whereas in other countries, such as the Netherlands, there is a nationwide program^7^. Various solutions could improve participation rates. As in the Netherlands where participation rate was 71,3%^7^, we could perform a more centralised screening program, distribute tests by mail, and also edit a national standard colonoscopy report. It has been shown that mailing the test to non-responders (after a first invitation), raised the compliance rate to the test^11^. Finally, another approach is the distribution of the test by more specialists, including GI, which is currently being investigated in France.

After a positive FIT, 70,5% of invitees underwent a colonoscopy and 62,5% had available reports (both colonoscopy and pathology). The number of individuals performing a colonoscopy without available reports in our database is unknown. We also found that mean time to colonoscopy after a positive test was 74,5 days when French guidelines recommend 31 days. This delay could be one explanation to the low colonoscopy participation rate in our population. There is also a need for improvement of colonoscopy performance rates.

With FIT in our population, PPV for AA and CRC were 30,5% and 8,5% respectively. These first results are what expected with FIT.

Another limit in the French CRC screening program is the definition used for “polyps at risk of transformation”. Indeed, guidelines no longer consider the villous component as a risk factor but this criterion is still used in our database^12^. Similarly, festooned polyps are not screened although they are now considered at risk of transformation when their size is 10 mm or larger, and/or histology shows dysplasia. Finally, hyperplasic polyps with size 10 mm or larger or localised outside of the rectum or sigmoid, should also benefit from an endoscopic surveillance.

In our population, we note that participation rates are still low but PPV for colorectal lesions are in range of results expected with FIT. In total, there is a space for improvement in the Parisian screening CRC program with FIT.
